# Budget-Aware Rescue Routing for Low-Overlap Indoor RGB-D Point Cloud Registration

**DOI:** 10.3390/s26102917

**Published:** 2026-05-07

**Authors:** Yingcheng Lin, Yizong Zhang, Junbo Liu, Jingyao Luan, Changlong Gao, Fang Yan

**Affiliations:** 1 The School of Microelectronics and Communication Engineering, Chongqing University, Chongqing 400044, China; linyc@cqu.edu.cn (Y.L.); 202412021068t@stu.cqu.edu.cn (Y.Z.); 2 Electric Power Research Institute of State Grid Jilin Electric Power Co., Ltd., Changchun 130021, China; 13894819779@139.com (J.L.); 18686687159@163.com (J.L.); gao18584311031@163.com (C.G.)

**Keywords:** indoor RGB-D sensing, low-overlap point cloud registration, budget-aware rescue routing, hardest-tail failures, runtime budget, deployable trade-off, Redwood, public-transfer validation

## Abstract

Low-overlap indoor RGB-D point cloud registration remains vulnerable to hard failures because robust recovery and deployment latency are rarely achieved by one registrar. We present a budget-aware rescue-routing framework that keeps PointDSC+FCGF as the fast primary path and separates deployable pre-rescue gates from frozen-candidate selector analysis. On 3DLoMatch, the frozen selector DRACO-Stack reaches strict success of 0.5205 vs. 0.4278 for PointDSC+FCGF, while the deployable DRACO-Gate reaches 0.4801; a matched accuracy-only CoFiNet reconstruction on the same 1781 pairs reaches 0.5390. On 3DMatch, DRACO-Route activates 369/1623 pairs and reaches 0.8885 at 721.18 ms, compared with 0.8694 for PointDSC+FCGF and 0.9082 at 8310.48 ms for always-on RegTR. Redwood is used as public-transfer validation, where PointDSC+FCGF reaches 0.1425 vs. 0.1043 for PointDSC+FPFH. The results support selective indoor hard-tail rescue under an explicit runtime budget, without claiming a universal scene-free router or a new backbone.

## 1. Introduction

Point cloud registration is central to indoor RGB-D mapping, reconstruction, localization, and scene understanding. Classical ICP-, FPFH/FGR-, and TEASER++ -style pipelines remain important references under partial overlap [1,2,3,4], while 3DMatch, 3DLoMatch, and Redwood provide standard RGB-D evaluation settings [5,6,7]. Recent surveys and learned registrars, including FCGF, D3Feat, Predator, PointDSC, GeoTransformer, and RegTR, have improved correspondence quality and low-overlap robustness [6,8,9,10,11,12,13,14,15]. A deployment gap remains: the models that rescue the hardest pairs are often too expensive to run on every pair in latency-bounded RGB-D systems.

This study addresses that deployment gap rather than proposing another backbone. It asks whether a fast default path can be retained for most pairs while heavier rescue is invoked only when route metadata indicate concentrated failure risk. The contribution is therefore an external policy layer over fixed registrars, not a new feature extractor, a jointly trained mixture-of-experts model, or an oracle selector. The work is related to selective computation and conditional inference, but here, the decision unit is a point–cloud pair, and the budget is measured directly in registration latency [16,17,18].

The frozen protocol supports this framing on three benchmarks. On 3DLoMatch, DRACO-Gate improves strict success from 0.4278 to 0.4801 using primary-run cues, and the frozen selector DRACO-Stack reaches 0.5205 over already materialized candidates. A matched accuracy-only CoFiNet reconstruction reaches 0.5390 on the same 1781 pairs, placing the selector near a strong recent baseline without treating it as a new backbone. On 3DMatch, DRACO-Route improves strict success from 0.8694 to 0.8885 at 721.18 ms, whereas always-on RegTR reaches 0.9082 at 8310.48 ms. Redwood is retained as public-transfer validation: PointDSC+FCGF improves over PointDSC+FPFH, but the routed Redwood probe is marginal and unstable under cross-validation.

This study makes three focused contributions: a budget-aware routing framework that keeps PointDSC+FCGF as the fast path and invokes rescue only for bounded difficult subsets; a reproducible routing audit with retained features, thresholds, activation counts, rescued/regressed counts, and paired exact tests; and evidence that gains are concentrated in hard-tail scenes. The method is therefore targeted failure recovery under an accuracy—latency budget, while Redwood routing and outdoor/LiDAR transfer remain limitations.

## 2. Related Work and Positioning

### 2.1. Point–Cloud Registration Backbones

Classical rigid registration combines geometric descriptors, correspondence generation, robust global alignment, and local refinement. ICP, FPFH/FGR, and TEASER++ remain core references but depend strongly on initialization, descriptor distinctiveness, and overlap quality [1,2,3,4]. Learned descriptors and registration systems such as FCGF, D3Feat, Predator, PointDSC, DCP, RPM-Net, DGR, and SpinNet improve correspondence quality, while transformer, partial-overlap, and coarse-to-fine systems such as OMNet, Lepard, GeoTransformer, CoFiNet, SC2-PCR, RoITr, and RegTR improve reasoning about overlap and global structure [6,11,12,13,14,15,19,20,21,22,23,24,25,26,27]. These methods define the backbone context; the present work instead examines how fixed registrars can be allocated under a runtime budget.

Low-overlap indoor RGB-D benchmarks make this allocation view important: a slow registrar may rescue a difficult subset, whereas a fast default path may already solve most pairs but fail on a hard tail. We therefore treat backbones as available components and ask when the system should pay for the heavier one. Future primary or rescue models could be inserted into the same framework.

### 2.2. Selective Computation and Routing

The policy layer is related to selective computation, conditional inference, uncertainty-aware abstention, and mixture-of-experts models [16,17,18]. Unlike token or classification routing, the decision unit here is a point–cloud pair, the output is a rigid transformation, and the cost is measured as registration runtime. Consequently, route activation, recovered failures, regressions, and missed rescue needed pairs are more informative than a single mean accuracy value.

This distinction motivates the separation between deployable triggers and frozen-candidate analysis. A valid low-cost trigger may use primary-path metadata, self-consistency cues, and scene metadata available before rescue; candidate-side deltas are reserved for auditing already materialized outputs. Thus, the 3DMatch scene bundle is the clearest deployable route, the 3DLoMatch selector family is an accuracy—runtime frontier over frozen candidates, and Redwood routing remains diagnostic.

### 2.3. Baseline Positioning Under Frozen Protocols

Recent low-overlap papers report strong 3DLoMatch results, including attention-based, buffer-style, transformer, and geometry-color fusion variants [28,29,30]. Because cross-paper comparisons depend on descriptors, pair filtering, thresholds, preprocessing, and hardware, the tables separate matched frozen rows from the literature context. The claim is not that the routed system is a universal state-of-the-art backbone, but that selective rescue yields a useful operating point between a fast primary baseline and expensive, always-on rescue.

## 3. Materials and Methods

### 3.1. Problem Formulation

Let an input pair be denoted by

$$x_{i}=\left(P_{i}^{s},P_{i}^{t}\right),$$

where $P_{i}^{s}$ and $P_{i}^{t}$ are the source and target point clouds derived from RGB-D sensing data. The registration target is a rigid transformation

$${\hat{T}}_{i}\in SE\left(3\right)$$

that aligns $P_{i}^{s}$ to $P_{i}^{t}$.

The primary registrar is denoted by $f_{p}$, which produces a preliminary estimate ${\hat{T}}_{i}^{\left(p\right)}=f_{p}\left(x_{i}\right)$. Route metadata are then extracted from the primary pass as

$$m_{i}=\phi \left(x_{i},{\hat{T}}_{i}^{\left(p\right)}\right),$$

where ϕ(·) summarizes correspondence statistics, confidence-related counts, self-consistency signals, and lightweight runtime proxies available without invoking the heavy rescue branch. For benchmark *b*, a routing policy $\pi_{b}\left(m_{i}\right)\in \left\{0,1\right\}$ decides whether the final output should remain on the primary path or be replaced by a benchmark-specific rescue output $f_{r}^{\left(b\right)}\left(x_{i}\right)$:

$${\hat{T}}_{i}=\left\{\begin{array}{cc} {\hat{T}}_{i}^{\left(p\right)}, & \pi_{b}\left(m_{i}\right)=0,\\ f_{r}^{\left(b\right)}\left(x_{i}\right), & \pi_{b}\left(m_{i}\right)=1. \end{array}\right.$$

This notation makes explicit that the contribution is an external decision layer over fixed registrars rather than a jointly trained expert model.

The evaluation uses a strict success indicator

$$s_{i}=1\;\left({\mathrm{RE}}_{i}\leq 5^{\circ }\;\wedge \;\frac{{\mathrm{TE}}_{i}}{{\mathrm{diag}}_{i}}\leq 0.05\right),$$

where ${\mathrm{RE}}_{i}$ is rotation error, ${\mathrm{TE}}_{i}$ is translation error, and ${\mathrm{diag}}_{i}$ is the benchmark-specific scene diagonal used for normalization. A relaxed pass criterion is also reported:

$$p_{i}=1\;\left({\mathrm{RE}}_{i}\leq 10^{\circ }\;\wedge \;\frac{{\mathrm{TE}}_{i}}{{\mathrm{diag}}_{i}}\leq 0.10\right).$$

For a routed system on benchmark *b* with rescue invocation rate $\rho_{b}$, the expected runtime can be summarized as

$$E\left[t|b\right]=t_{p}+\rho_{b}\;\left(t_{r}^{\left(b\right)}-t_{p}\right),$$

where $t_{p}$ is the primary-path runtime, and $t_{r}^{\left(b\right)}$ is the rescue runtime under benchmark-specific policy $\pi_{b}$. This form is useful because the objective of the framework is not unconstrained accuracy maximization, but bounded recovery of difficult failures.

### 3.2. Primary Path and Rescue Paths

The default path in the promoted results is PointDSC+FCGF, which combines PointDSC with FCGF descriptors [11,13]. Within the synchronized frozen protocol, it is the strongest, directly audited, low-cost starting point. Predator and D3Feat were not rerun here as matched cheap-primary rows; accordingly, the manuscript does not claim that this default choice is globally optimal. The strongest heavy rescue used in the promoted 3DMatch route is RegTR [15], which provides stronger hard-tail recovery but incurs substantially higher latency when invoked on every pair. The comparison space also includes PointDSC+FPFH, Official 3DMatch, Open3D ICP, Open3D FGR+ICP, and TEASER++ + FPFH as reference baselines [1,2,3,4,5].

### 3.3. Routing Signals and Policies

Table 1 summarizes the cue specification used in the revised manuscript. We distinguish pre-routing signals, which are available after the primary pass and before rescue execution, from post hoc candidate quantities, which are informative for frozen-candidate analysis but cannot by themselves justify a low-cost trigger. This distinction is essential because a routed system should not first execute a heavy candidate and only then decide whether that candidate should have been invoked.

Table 2 summarizes the policy instances retained after revision. The table is intentionally explicit about which policies are admissible before rescue and which policies use candidate-side information. On 3DLoMatch, DRACO-Bundle and DRACO-Gate are deployable pre-rescue gates because they use only primary-trace counts, ratios, and scene metadata. DRACO-ET and DRACO-Stack are frozen-candidate selectors: they are useful for measuring complementarity among saved candidates, but they are not claimed to be stand-alone low-cost triggers. On 3DMatch, the promoted route is deliberately simpler: pairs from studyroom2 and erika are sent to RegTR, whereas all remaining scenes stay on PointDSC+FCGF. On Redwood, the scene-gate probe is reported only as complementary transfer evidence because the deployable gain is marginal.

### 3.4. Threshold Selection and Decision Audit

Route thresholds are fixed before final evaluation and are not tuned on qualitative examples. Counts and runtimes use native units, ratio cues use their native [0,1] range, and no extra scaling is applied. The 3DLoMatch deployable gates use corr_count, predicted_positive, and predicted_positive_ratio; selector rows additionally use saved candidate-side probabilities, margins, and delta/ratio features, so they are labeled frozen-candidate analysis. The retained selector margin is 0.01. For 3DMatch, all studyroom2 and erika pairs—the routed hardtail369 bundle—are sent to RegTR, while all other scenes stay on PointDSC+FCGF. The Redwood diagnostic scene gate uses the thresholds shown in Table 2.

The decision audit uses four pair-level categories: rescued, regressed, missed rescue needed, and unchanged. This is necessary because selective rescue can increase average success while still regressing easy pairs, and high activation can mask poor decision precision. The experiments therefore report strict success, runtime, activation rate, rescued/regressed counts, and scene locations of missed cases. Supplementary Note S4 also records two admissible 3DMatch feature-group audits; both are more recall-oriented but too latency-intensive, so the promoted 3DMatch row remains the transparent scene bundle rather than a learned generic router.

### 3.5. Budget-Aware Inference Procedure

At the inference time, PointDSC+FCGF first generates route metadata. Unflagged pairs retain the primary transform, whereas flagged pairs use the assigned rescue branch. The procedure remains external to the registrars and is therefore a deployment-oriented system layer rather than a new end-to-end network.

## 4. Experimental Setup

### 4.1. Benchmarks and Frozen Pair Pools

The evaluation uses three public RGB-D benchmarks with fixed roles: 3DLoMatch as the 1781-pair low-overlap stress benchmark, 3DMatch as the 1623-pair indoor deployment benchmark with a hardtail369 subset, and Redwood as a 1256-pair public transfer check with a deterministic 128-pair geometry anchor [5,6,7]. KITTI is retained only as supplementary limitation evidence [31]. Fixed pair CSVs and exact file names are provided in the package manifest.

### 4.2. Baselines, Metrics, and Runtime Accounting

The comparison set includes PointDSC+FCGF, PointDSC+FPFH, RegTR, Official 3DMatch, Open3D ICP, Open3D FGR+ICP, and TEASER++ + FPFH [32]. We report strict success, pass@10/0.10, median rotation error, median normalized translation error, and median runtime. Strict success is the primary endpoint, while runtime is a co-equal deployment metric.

### 4.3. Implementation Details and Reproducibility Assets

The release assets record the following compute environment: 36 logical CPU cores, 18 physical CPU cores, 20.88 GB available RAM, and an NVIDIA GeForce GTX 1080 Ti GPU. The synchronized package also includes benchmark-preparation scripts, route-training utilities, figures, manifests, generated summary tables, and a single row-level SQLite evidence database, revision_experiment_data.sqlite. The database stores 18,798 routed per-pair rows, 1781 matched CoFiNet per-pair rows, paired-significance tables, policy activation/regression audits, runtime distributions, routing-feature thresholds, missing-resource notes, and 47 validation checks. The main indoor rows should therefore be interpreted as deterministic benchmark scores from a frozen evaluation bundle rather than as results that depend on renewed route sweeps or ad hoc local reruns.

The package is organized around frozen identifiers rather than transient local paths. Each main table traces to a retained per-pair summary or scene-level aggregation, and the assets preserve route family, margin, split, and rescue candidate. Pair pools and success definitions are fixed before aggregation.

Runtime is part of the experimental outcome. Promoted rows report median per-pair runtime under the same recorded environment, and Supplementary Note S1 adds mean, standard deviation, quartiles, and 95th-percentile latency. The manuscript therefore emphasizes operating points rather than accuracy alone.

## 5. Results

### 5.1. Main Benchmark Comparison

Table 3 organizes the evidence into three roles: 3DLoMatch for low-overlap recovery, 3DMatch for deployable indoor accuracy—latency trade-offs, and Redwood for public-transfer validation. DRACO-Stack reaches 0.5205 strict success on 3DLoMatch, versus 0.4278 for PointDSC+FCGF and 0.5390 for the matched accuracy-only CoFiNet row. On 3DMatch, DRACO-Route reaches 0.8885 at 721.18 ms, whereas always-on RegTR reaches 0.9082 at 8310.48 ms. On Redwood, PointDSC+FCGF reaches 0.1425 vs. 0.1043 for PointDSC+FPFH.

Table 4 separates matched evidence from literature context. CoFiNet is included because saved trajectory logs cover the full 1781-pair pool, but its runtime was unrecoverable. GeoTransformer and Predator remain literature-context rows because the frozen workspace contained only smoke-level GeoTransformer outputs and incomplete Predator transformations.

### 5.2. 3DLoMatch Accuracy-Efficiency Trade-Off

**Figure 1:**
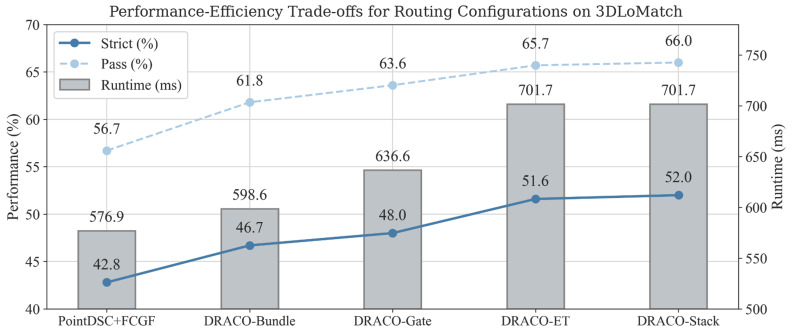
3DLoMatch strict/pass success and median-runtime trade-off across the primary baseline, deployable gates, and frozen-candidate selectors. Bars denote median runtime, and the right-hand runtime axis is separated from point annotations for readability.

The 3DLoMatch operating points separate deployable gates from frozen-candidate selectors. Figure 1 and Table 5 and Table 6 summarize the corresponding accuracy–runtime trade-off and paired significance tests. DRACO-Bundle and DRACO-Gate use only primary-trace counts, ratios, and scene metadata, whereas DRACO-ET and DRACO-Stack are selector analyses over saved candidates. DRACO-Stack improves strict success by +0.0926 over PointDSC+FCGF with exact paired $p=8.30\times 10^{-39}$ (177 rescued, 12 regressed), and DRACO-Gate improves by +0.0522 with exact paired $p=7.75\times 10^{-11}$ (150 rescued, 57 regressed).

### 5.3. Scene-Level Distribution of the 3DLoMatch Gains

**Figure 2:**
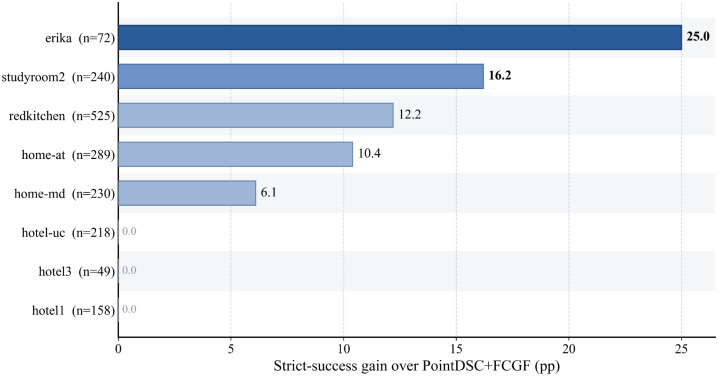
3DLoMatch scene-level strict-success gains of the current-best routed selector relative to PointDSC+FCGF. Darker blue indicates a larger positive gain, whereas lighter blue indicates a smaller positive gain; zero-gain scenes are shown without visible bars. The most visible gains occur on erika and studyroom2.

The 3DLoMatch gains are scene-concentrated. Figure 2 and Table 7 show the largest gains in erika, studyroom2, and redkitchen; smaller gains in home_at and home_md; and no measurable gain in the hotel scenes. The route therefore targets specific failure modes rather than broad average-case superiority.

### 5.4. 3DMatch Hardest-Tail Analysis and Route Activation

**Figure 3:**
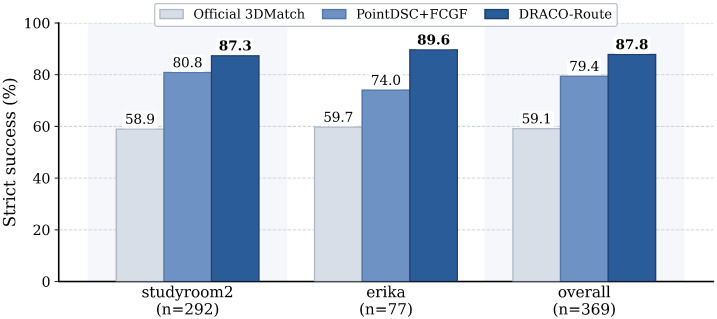
Strict-success comparison on the hardest 3DMatch subset. The gain is concentrated on studyroom2 and erika, which are exactly the scenes targeted by the routed policy.

On the hardtail369 subset, Table 8 and Figure 3 show that PointDSC+FCGF reaches 0.7940 strict success, whereas both DRACO-Route and always-on RegTR reach 0.8780. They coincide here because every pair belongs to a routed scene; the full-benchmark runtime advantage appears only because the other 1254 pairs stay on PointDSC+FCGF.

Table 9 shows that the 22.74% routed fraction is concentrated entirely in studyroom2 and erika. Across the full benchmark, the route rescues 39 pairs and regresses on 8; all regressions occur in studyroom2. Supplementary Note S1 reports the route-rate sweep, runtime audit, and paired significance (exact two-sided $p=5.54\times 10^{-6}$).

**Figure 4:**
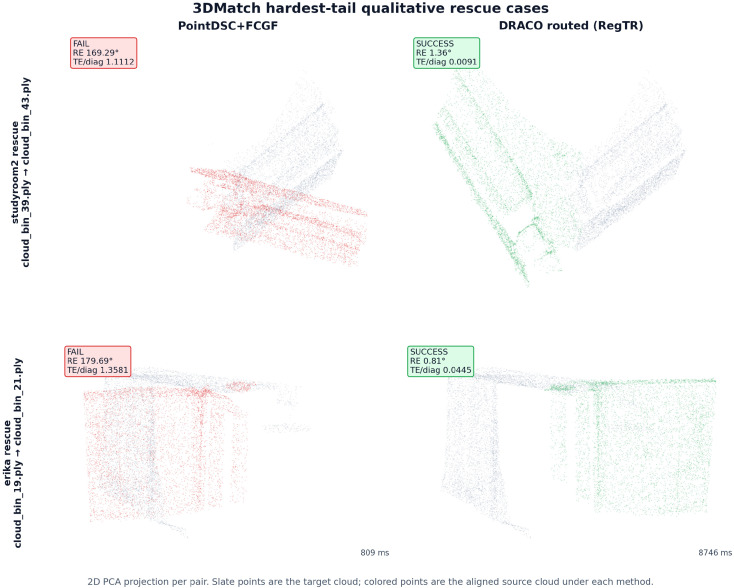
Qualitative hardest-tail rescue cases. The routed policy switches from PointDSC+FCGF to RegTR and recovers strict success on representative studyroom2 and erika pairs.

Figure 4 and Table 10 add the requested negative boundary cases: routed regression, missed rescue, unchanged failure, net-negative always-on expansion avoided by bounded routing, and a hotel-family minimal-effect case. Thus, the failure boundary is explicit rather than hidden behind aggregate gains.

### 5.5. Redwood Public-Transfer Validation

Redwood is retained as a transfer-validation benchmark, not as a routed headline benchmark. Table 11 separates the promotable transfer row from diagnostics: PointDSC+FCGF improves strict success from 0.1043 to 0.1425 relative to PointDSC+FPFH, whereas the deployable scene_gate adds only +0.0024 and has a lower five-fold cross-validated mean than the baseline. The Redwood claim therefore rests on the transfer of the primary descriptor stack, not routed superiority.

## 6. Discussion

The results support a narrow deployment claim: rescue routing helps when extra model capacity is invoked only where failure risk is concentrated. On 3DLoMatch, the gain comes from a small subset of scenes; on 3DMatch, the route recovers much of the hard-tail gap while leaving 1254 of 1623 pairs on the fast primary path. Thus, the method is an operating-point adjustment for indoor RGB-D sensing systems, not a universal recipe for combining registrars.

The route must be judged by both gain and error profile. The conservative 3DMatch policy switches only 369 pairs, keeping runtime closer to PointDSC+FCGF than to always-on RegTR, but it also leaves rescue-needed cases outside the routed scenes unresolved.

The 3DLoMatch selector family likewise shows useful but uneven complementarity: erika, studyroom2, redkitchen, and home-at benefit, whereas hotel scenes show no measurable strict-success gain. The qualitative boundary cases and the eight 3DMatch regressions make this limitation explicit.

Redwood clarifies the transfer boundary. The promotable evidence is the PointDSC+FCGF transfer row, while the Redwood scene gate is diagnostic because its gain is only +0.0024 and its cross-validated mean falls below the baseline. The balanced128 Open3D FGR+ICP row is a geometry-only reference, and KITTI remains supplementary limitation material.

Several limitations follow. The promoted 3DMatch route is benchmark-aware because it keys on scene identity, so it is a scene-aware operating point rather than a universal uncertainty router. The stronger 3DLoMatch methods in Table 4 are literature-context rows, not matched-protocol reruns. Runtime is environment-specific and right-skewed under selective rescue; Supplementary Note S1 reports the mean, standard deviation, quartiles, and 95th-percentile latency. No small neural router is promoted here; richer learned routing remains future work. Finally, the evidence is limited to indoor static RGB-D pairs, and outdoor scenes, moving robots, and RGB-D + LiDAR fusion require additional cues and validation.

## 7. Conclusions

Within a frozen low-overlap indoor RGB-D protocol, budget-aware rescue routing improves hard-tail registration while preserving a practical runtime envelope. The strongest evidence is the 3DLoMatch gain and the 3DMatch deployable operating point, where limited rescue closes much of the hard-tail gap without always-on RegTR. Redwood validates the transfer of the primary descriptor stack, while KITTI remains supplementary limitation material. The contribution is an external routing strategy over fixed registrars, not a new backbone or proof of universal routing. Future work should replace benchmark-aware priors with uncertainty-driven rescue signals and real-time RGB-D validation.

## Figures and Tables

**Table 1:** Routing cue families retained in the revision. Supplementary Note S4 reports the exact raw field names and thresholds.

Cue Family	Representative Cues	Pre-Rescue?	Role in This Study
Primary trace	Correspondence count; predicted-positive count and ratio; matching, inference, and runtime terms	Yes	Low-cost admissible cues for the retained 3DLoMatch gates and admissible 3DMatch probe audit.
Self-consistency	Inlier score; mean/RMSE consistency; trimmed-mean ratio; hybrid score summaries	Yes	Additional low-cost cues used in the self-augmented audit and frozen 3DLoMatch selector analysis.
Scene metadata	Benchmark name, scene label, and scene key	Yes	Indexes the retained 3DLoMatch scene rules and the promoted 3DMatch studyroom2 + erika bundle.
Candidate-side deltas	Candidate confidence, selector margin, candidate-vs-primary delta/ratio terms, and candidate runtime terms	No	Used only for frozen-candidate selector analysis; not a trigger before rescue.

Note: Native units are used throughout. Full field names, normalization status, and saved thresholds are reported in Supplementary Note S4.

**Table 2:** Policy-specific routing rules, feature timing, and pair-level audit. Counts, ratios, and runtimes are used in native units with no additional scaling.

Variant	Class/Timing	Retained Rule/Threshold	Pair-Level Audit
DRACO-Bundle	Deployable gate; pre-rescue	Scene PPR gates:redkitchen <0.07;studyroom2 <0.05;erika <0.06.	1356/1781 activated;127 rescued/40 regressed;net +87.
DRACO-Gate	Deployable gate; pre-rescue	Saved primary-trace rules.Example:studyroom2 → RegTR if PPR <0.06;redkitchen → PredatorBundle if PP ≤300 and CC ≤8000.	790/1781 activated;150 rescued/57 regressed;net +93.
DRACO-ET	Frozen selector; post-cand.	Extra-Trees selector over saved candidates;600 trees; five pair-hash folds; global margin 0.01 plus grouped overrides.	931 non-primary selections;177 rescued/20 regressed;net +157.
DRACO-Stack	Frozen selector; post-cand.	Stack overlay aboveDRACO-ET;replace the incumbent only when the overlay score exceeds it by margin 0.01.	8 overlay updates over DRACO-ET; 177 rescued/12 regressed vs. primary;net +165.
DRACO-Route	Deployable scene bundle; pre-rescue	3DMatch scene key in {studyroom2, erika} → RegTR;all other scenes stay on PointDSC+FCGF.	369/1623 activated;39 rescued/8 regressed;net +31.
Redwood scene	Diagnostic gate; pre	livingroom2-simulated:PPR <0.03 and PP ≤200;office1-simulated:PPR <0.04.	+0.24 pp over Redwood primary;diagnostic only.

Note: PPR = predicted_positive_ratio, PP = predicted_positive, and CC = corr_count. For DRACO-Stack, the “8 overlay updates” are changes relative to DRACO-ET, not eight total pairs compared with the primary. Rescued/regressed counts are always computed pairwise against PointDSC+FCGF.

**Table 3:** Main benchmark comparison across the synchronized indoor and transfer benchmarks. Strict success is the primary endpoint; pass@10/0.10 is the relaxed success criterion. The CoFiNet row is a same-pool accuracy reconstruction without recovered runtime. Redwood is reported here through the frozen PointDSC+FCGF transfer row and fixed baselines only; complementary routed Redwood diagnostics are reported separately in the Redwood public-transfer validation subsection, and KITTI remains supplementary.

Benchmark	Method	Pairs	Strict	Pass	RE	TE Ratio	Time (ms)
3DLoMatch	CoFiNet-matched	1781	**0.5390**	0.7075	4.2647	0.0285	—
	DRACO-Stack	1781	0.5205	0.6597	4.3983	0.0352	701.74
	DRACO-ET	1781	0.5160	0.6569	4.4379	0.0359	701.74
	DRACO-Gate	1781	0.4801	0.6356	4.8543	0.0394	636.57
	DRACO-Bundle	1781	0.4767	0.6232	4.9208	0.0397	649.22
	PointDSC+FCGF	1781	0.4278	0.5665	5.8045	0.0485	576.88
3DMatch	RegTR	1623	**0.9082**	0.9433	1.4378	0.0104	8310.48
	DRACO-Route	1623	0.8885	0.9316	1.6262	0.0118	721.18
	PointDSC+FCGF	1623	0.8694	0.9230	1.7947	0.0127	636.82
	Official 3DMatch	1623	0.7135	0.7905	1.9123	0.0134	—
	PointDSC+FPFH	1623	0.6870	0.7388	2.1704	0.0160	646.13
	Open3D ICP	1623	0.0006	0.0265	30.7853	0.1836	803.30
	TEASER++ FPFH	1623	0.0006	0.0049	59.7448	0.3359	1647.32
	Open3D FGR+ICP	1623	0.0000	0.0055	58.7034	0.3785	1139.15
Redwood	PointDSC+FCGF	1256	**0.1425**	0.1839	91.0365	0.4371	1196.10
	PointDSC+FPFH	1256	0.1043	0.1298	96.9693	0.4494	1143.83
	FGR+ICP-128	128	0.0000	0.0000	108.1122	0.6191	3823.34

Note: Bold values indicate the highest strict success within each benchmark block.

**Table 4:** 3DLoMatch matched baseline and literature context. Matched rows use the same 1781-pair pool and strict criterion; literature rows are not treated as direct runtime comparisons.

Method	Strict/Recall (%)	Evidence Class in This Paper	Used for Direct Ranking
CoFiNet-matched [25]	53.90	Same 1781-pair pool; saved trajectory reconstruction; runtime unavailable.	Accuracy only
DRACO-Stack	52.05	Same 1781-pair pool; frozen-candidate selector over saved candidates.	Yes
Predator [6]	54.2	Original paper context; workspace output incomplete for a matched rerun.	No
CoFiNet original [25]	67.5	Literature context from upstream report.	No
GeoTransformer [14]	75.0	Literature context; only smoke-level outputs found in the frozen workspace.	No
SC2-PCR [26]	57.8/69.5	Original paper context; settings differ, and no full saved run available.	No
RoITr [27]	74.7	Original paper context only.	No

**Table 5:** 3DLoMatch operating-point audit. “Act./sel.” means pre-rescue activations for deployable gates, non-primary selections for DRACO-ET, and overlay updates over DRACO-ET for DRACO-Stack. The reference method for ΔS is PointDSC+FCGF.

Method	Policy Class	Act./Sel.	Strict	Res./Reg.	Median ms	Mean/P95 ms
DRACO-Stack	Frozen selector	8 overlay	**0.5205**	177/12	701.74	2853/17,041
DRACO-ET	Frozen selector	931 selected	0.5160	177/20	701.74	2853/17,041
DRACO-Gate	Deployable gate	790 routed	0.4801	150/57	636.57	1626/9577
DRACO-Bundle	Deployable gate	1356 routed	0.4767	127/40	649.22	629/1155
PointDSC+FCGF	Primary baseline	0	0.4278	0/0	576.88	559/1084

Note: Bold value indicates the highest strict success in this operating-point audit.

**Table 6:** 3DLoMatch paired significance against PointDSC+FCGF in the synchronized 1781-pair pool.

Policy	Class	Success	Rescued	Regressed	*p* Value
DRACO-Bundle	Deployable	0.4767	127	40	$9.45\times 10^{-12}$
DRACO-Gate	Deployable	0.4801	150	57	$7.75\times 10^{-11}$
DRACO-ET	Frozen selector	0.5160	177	20	$1.32\times 10^{-32}$
DRACO-Stack	Frozen selector	0.5205	177	12	$8.30\times 10^{-39}$

**Table 7:** 3DLoMatch scene-level strict-success gains for the current-best routed selector relative to PointDSC+FCGF.

Scene	Pairs	Primary	Routed	ΔS
erika	72	0.3056	0.5556	**0.2500**
studyroom2	240	0.3208	0.4833	**0.1625**
redkitchen	525	0.4743	0.5962	**0.1219**
home-at	289	0.3737	0.4775	**0.1038**
home-md	230	0.4043	0.4652	**0.0609**
hotel-uc	218	0.5826	0.5826	0.0000
hotel1	158	0.3987	0.3987	0.0000
hotel3	49	0.4694	0.4694	0.0000

Note: Bold values indicate positive strict-success gains over the primary baseline; zero-gain rows are not bolded.

**Table 8:** 3DMatch hardtail369 comparison.

Method	Pairs	Strict	RE	TE Ratio	Time (ms)
Official 3DMatch	369	0.5908	2.1813	0.0177	—
PointDSC+FCGF	369	0.7940	2.0822	0.0180	700.14
DRACO-Route	369	**0.8780**	1.4043	0.0113	8521.73
RegTR	369	**0.8780**	1.4043	0.0113	8521.73

Note: Bold values indicate the highest strict success in this hardtail comparison; equal bold values denote a tie.

**Table 9:** Scene-wise 3DMatch route audit for the promoted fixed bundle.

Scene Slice	Routed/Total	Route %	Rescued	Regressed	Missed Rescue Needed
studyroom2	292/292	100.0	27	8	0
erika	77/77	100.0	12	0	0
Other scenes	0/1254	0.0	0	0	70
**Overall**	**369/1623**	**22.74**	**39**	**8**	**70**

Note: The bold row is the aggregate total across all scene slices. The routed fraction is concentrated entirely in studyroom2 and erika. All eight regressions occur in studyroom2, whereas erika contains none. The paired primary-vs-routed comparison yields 39 rescued pairs, 8 regressed pairs, and an exact two-sided $p=5.54\times 10^{-6}$.

**Table 10:** Representative qualitative boundary cases from the pair-level audit.

Case Type	Scene	Pair	Primary RE/TE	Selected RE/TE	Interpretation
Successful rescue	studyroom2	cloud_bin_8 → cloud_bin_51	10.91/0.0526	0.65/0.0030	Primary fails but routed RegTR succeeds.
Routed regression	studyroom2	cloud_bin_36 → cloud_bin_39	2.58/0.0342	92.72/0.0432	A correct primary pose is replaced by a failed rescue.
Missed rescue	redkitchen	cloud_bin_11 → cloud_bin_28	85.32/0.6501	85.32/0.6501	The route stays on primary, although always-on RegTR would have rescued the pair.
Unchanged failure	redkitchen	cloud_bin_15 → cloud_bin_21	117.87/0.5541	117.87/0.5541	Both the routed output and rescue counterfactual remain failures.
Net-negativeexpansion avoided	redkitchen	cloud_bin_0 → cloud_bin_32	4.14/0.0141	4.14/0.0141	Bounded routing avoids an always-on rescue failure.
Minimal-effectscene	hotel-uc	cloud_bin_0 → cloud_bin_1	1.57/0.0112	1.57/0.0112	Hotel-family scene with little rescue benefit.

**Table 11:** Redwood transfer comparison and complementary routed diagnostics. Only the frozen PointDSC+FCGF row and its fixed baselines support the main-table transfer claim; the scene-gate route is deployable but non-promotable, whereas rescue-on-failure and oracle are diagnostic upper bounds only.

Method	Role	Strict	Pass	Time (ms)	ΔS
PointDSC+FPFH	Alt. primary	0.1043	0.1298	1143.83	—
PointDSC+FCGF	Main transfer row	**0.1425**	0.1839	1196.10	0.0382
Scene Gate	Deployable route probe	0.1449	0.1847	1313.55	0.0024
Rescue On Failure	Upper bound	0.1616	0.1959	1205.98	0.0191
Oracle	Oracle ceiling	**0.1616**	0.2006	1659.27	0.0191

Note: Bold values indicate the highest strict success within the transfer/diagnostic comparison; equal bold values denote a tie.

## Data Availability

The public benchmark datasets are available from their original sources. The submitted reproducibility package provides revision_experiment_data.sqlite, route manifests, generated tables, and validation checks; the SQLite database stores the per-pair rows supporting all main results.

## Supplementary Materials

The following supporting information can be downloaded at: https://www.mdpi.com/article/10.3390/s26102917/s1. The supporting information includes Figure S1, strict-success comparison on the 3DMatch hardtail369 split; Figure S2, qualitative rescue cases from studyroom2 and erika; Supplementary Table S1, route-activation sensitivity and scene-wise route percentages; Supplementary Table S2, runtime-distribution and confidence-interval summaries; Supplementary Note S1, paired significance and route-rate analysis; Supplementary Note S2, qualitative success, regression, missed-rescue, and no-gain cases; Supplementary Note S3, Redwood public-transfer audit; and Supplementary Note S4, cue-level routing audits, matched CoFiNet evidence, missing-resource notes, and the SQLite reproducibility manifest.
