# Supplementary material for: Budget-Aware Rescue Routing for Low-Overlap Indoor RGB-D Point Cloud Registration
Source: Sensors (Basel). 2026 May 7;26(10):2917. doi: 10.3390/s26102917 (PMC13210820; doi:10.3390/s26102917)
Supplement: Supplementary file 1 [file sensors-26-02917-s001.zip › sensors-4261809-supplementary.pdf]

Supplementary Materials

Budget-Aware Rescue Routing for Low-Overlap Indoor RGB-D Point Cloud Registration

Yingcheng Lin, Yizong Zhang, Junbo Liu, Jingyao Luan, Changlong Gao and Fang Yan

Manuscript ID: sensors-4261809

Coverage Statement

This single supplementary file consolidates all items cited in the back matter of the revised manuscript: Figure S1, Figure S2, Supplementary Table S1, Supplementary Table S2, Supplementary Note S1, Supplementary Note S2, Supplementary Note S3, and Supplementary Note S4. Numeric entries are synchronized with the evidence database `revision_experiment_data.sqlite`; strict success uses the manuscript criterion RE $\leq$ 5 degrees and TE ratio $\leq$ 0.05 unless otherwise stated.

- **Figure S1**: strict-success comparison on the 3DMatch hardtail369 split.
- **Figure S2**: qualitative rescue cases from `studyroom2` and `erika`.
- **Supplementary Table S1**: route-activation sensitivity and scene-wise route percentages.
- **Supplementary Table S2**: runtime-distribution and Wilson confidence-interval summaries.
- **Supplementary Notes S1–S4**: paired significance and route-rate analysis; qualitative boundary cases; Redwood public-transfer audit; cue-level routing audits, matched CoFiNet evidence, missing-resource notes, and SQLite reproducibility manifest.

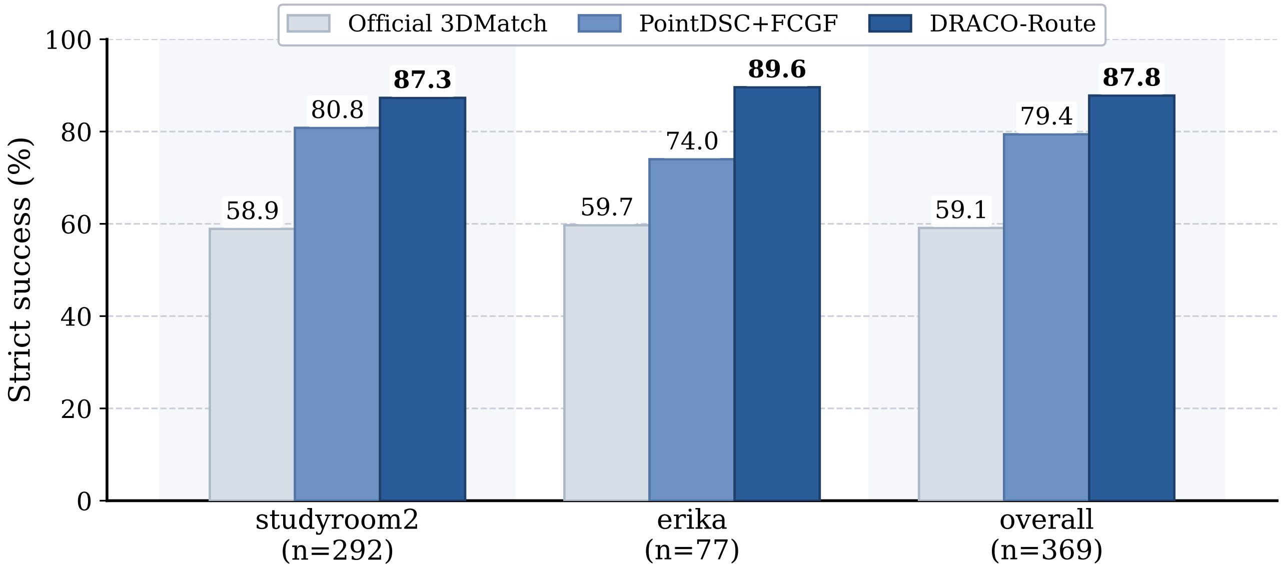

Figure S1: Strict-success comparison on the 3DMatch hardtail369 split. The hardtail369 subset is the fixed routed bundle `studyroom2` (292 pairs) plus `erika` (77 pairs), totaling 369 pairs. PointDSC+FCGF primary succeeds on 293/369 (79.40%); DRACO-Route and the RegTR counterfactual succeed on 324/369 (87.80%). The route rescues 39 primary failures and introduces 8 regressions on this subset.

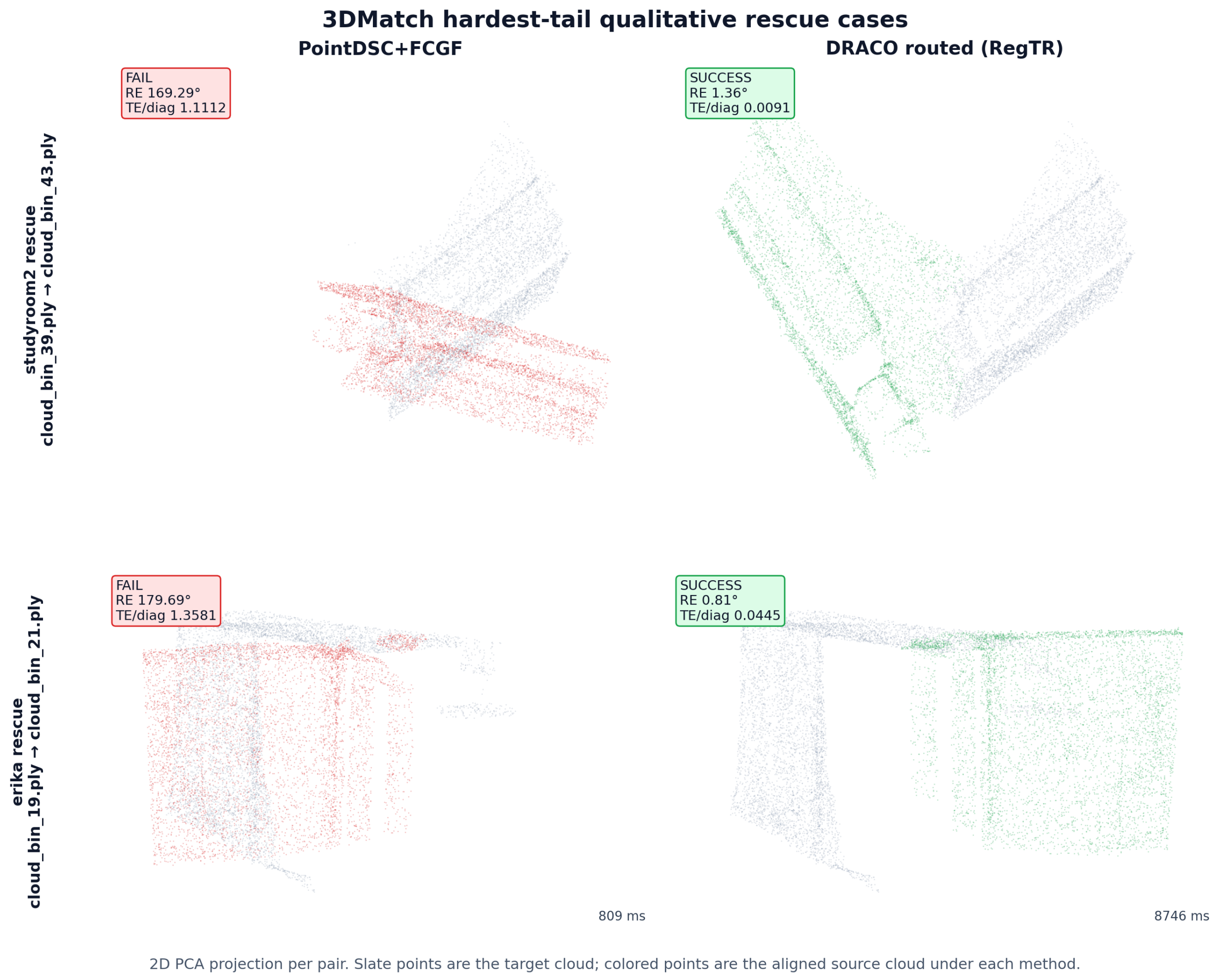

Figure S2: Qualitative rescue cases from `studyroom2` and `erika`. The routed policy switches from PointDSC+FCGF to RegTR on the fixed hardtail bundle and recovers representative strict-success cases.

Table S1: Route-activation sensitivity and scene-wise route percentages.

Panel	Scope	Rule or policy	Routed/ changed	Total	Route %	Success %	Rescued	Regressed	Missed	Net	Median ms	Notes
Panel A	3DMatch	Primary only	0	1623	0.00	86.94	0	0	–	0	636.82	3DMatch route-rate sensitivity
Panel A	3DMatch	Erika only -> RegTR	77	1623	4.74	87.68	12	0	–	12	647.39	3DMatch route-rate sensitivity
Panel A	3DMatch	Studyroom2 only -> RegTR	292	1623	17.99	88.11	27	8	–	19	701.00	3DMatch route-rate sensitivity
Panel A	3DMatch	Studyroom2 + Erika -> RegTR	369	1623	22.74	88.85	39	8	–	31	721.18	3DMatch route-rate sensitivity
Panel A	3DMatch	All pairs -> RegTR	1623	1623	100.00	90.82	109	46	–	63	8310.48	3DMatch route-rate sensitivity
Panel B	3DMatch: erika	Scene slice for promoted route	77	77	100.00	89.61	12	0	0	–	–	Scene-wise route percentage
Panel B	3DMatch: home_at	Scene slice for promoted route	0	156	0.00	94.87	0	0	3	–	–	Scene-wise route percentage
Panel B	3DMatch: home_md	Scene slice for promoted route	0	208	0.00	66.35	0	0	25	–	–	Scene-wise route percentage
Panel B	3DMatch: hotel1	Scene slice for promoted route	0	104	0.00	85.58	0	0	10	–	–	Scene-wise route percentage
Panel B	3DMatch: hotel3	Scene slice for promoted route	0	54	0.00	81.48	0	0	8	–	–	Scene-wise route percentage
Panel B	3DMatch: hotel_uc	Scene slice for promoted route	0	226	0.00	95.58	0	0	7	–	–	Scene-wise route percentage
Panel B	3DMatch: redkitchen	Scene slice for promoted route	0	506	0.00	95.45	0	0	17	–	–	Scene-wise route percentage
Panel B	3DMatch: studyroom2	Scene slice for promoted route	292	292	100.00	87.33	27	8	0	–	–	Scene-wise route percentage
Panel B	3DMatch: ALL	Scene slice for promoted route	369	1623	22.74	88.85	39	8	70	–	–	Scene-wise route percentage
Panel C	3DLoMatch	gate_threshold=0.05	436	1781	24.48	44.75	–	–	–	–	591.46	Saved deployable threshold-suite point
Panel C	3DLoMatch	gate_threshold=0.06	516	1781	28.97	44.97	–	–	–	–	595.66	Saved deployable threshold-suite point
Panel C	3DLoMatch	gate_threshold=0.07	575	1781	32.29	45.26	–	–	–	–	595.99	Saved deployable threshold-suite point
Panel C	3DLoMatch	scene_gate_full_fit_saved_rule	790	1781	44.36	48.01	–	–	–	–	636.57	Saved deployable threshold-suite point
Panel D	3DLoMatch	PointDSC+FCGF primary	0	1781	0.00	42.78	0	0	–	0	576.88	primary_baseline; frozen-candidate
Panel D	3DLoMatch	DRACO-Bundle	1356	1781	76.14	47.67	127	40	–	87	649.22	deployable_pre_rescue_gate; pre-rescue
Panel D	3DLoMatch	DRACO-Gate	790	1781	44.36	48.01	150	57	–	93	636.57	deployable_pre_rescue_gate; pre-rescue
Panel D	3DLoMatch	DRACO-ET	931	1781	52.27	51.60	177	20	–	157	701.74	frozen_candidate_selector; frozen-candidate; uses candidate-side features
Panel D	3DLoMatch	DRACO-Stack	8	1781	0.45	52.05	177	12	–	165	701.74	frozen_candidate_selector; frozen-candidate; uses candidate-side features

Note: For DRACO-ET and DRACO-Stack, changed counts are frozen-candidate selector changes rather than pre-rescue routed pairs. Missed rescue denotes primary failure where the rescue counterfactual would have succeeded but the promoted route stayed on primary.

Table S2: Runtime-distribution and Wilson confidence-interval summaries.

Benchmark	Policy	Total	Rt. rec.	Missing	Mean ms	SD ms	Median ms	Q1 ms	Q3 ms	P95 ms	Success %	Wilson 95% CI
3DLoMatch	DRACO-Bundle	1781	1781	0	628.88	322.47	649.22	312.74	856.72	1155.01	47.67	45.36-49.99
3DLoMatch	DRACO-ET	1781	1781	0	2853.39	5306.34	701.74	357.54	1028.45	17041.11	51.60	49.28-53.92
3DLoMatch	DRACO-Gate	1781	1781	0	1626.36	2906.80	636.57	281.15	916.37	9577.28	48.01	45.69-50.33
3DLoMatch	DRACO-Stack	1781	1781	0	2853.39	5306.34	701.74	357.54	1028.45	17041.11	52.05	49.73-54.36
3DLoMatch	PointDSC+FCGF primary	1781	1781	0	559.12	327.70	576.88	187.93	786.57	1083.55	42.78	40.51-45.10
3DMatch	DRACO-Route	1623	1619	4	2392.11	3438.48	721.18	444.96	1176.33	9586.41	88.85	87.22-90.29
3DMatch	PointDSC+FCGF primary	1623	1623	0	606.70	337.22	636.82	392.27	832.05	1115.00	86.94	85.21-88.49
3DMatch	RegTR counterfactual	1623	1619	4	8258.54	1612.49	8310.48	7358.04	9335.35	10766.87	90.82	89.32-92.13
Redwood	Redwood Oracle	1256	1256	0	1862.78	901.58	1659.27	1191.27	2335.97	3601.52	16.16	14.23-18.30
Redwood	Redwood PointDSC+FCGF primary	1256	1256	0	1268.99	422.45	1196.10	972.13	1452.05	2114.86	14.25	12.43-16.29
Redwood	Redwood Rescue On Failure	1256	1256	0	1293.59	469.46	1205.98	974.40	1471.07	2213.04	16.16	14.23-18.30
Redwood	Redwood Scene Gate	1256	1256	0	1527.81	728.53	1313.55	1069.77	1774.74	3017.26	14.49	12.65-16.55

Note: Right-skewed runtime is summarized by mean, standard deviation, quartiles, median, and 95th percentile. Success confidence intervals use Wilson intervals.

Supplementary Note S1. Paired significance and route-rate analysis

The promoted 3DMatch route invokes RegTR only for `studyroom2` and `erika`, routing 369/1623 pairs (22.74%). It improves strict success from 1411/1623 (86.94%) to 1442/1623 (88.85%) with 39 rescued pairs and eight regressions. The exact two-sided paired test over discordant pairs gives $p = 5.54 \times 10^{-6}$. Always-on RegTR remains more accurate but much slower, reaching 1474/1623 (90.82%) at a median runtime of 8310.48 ms.

On 3DLoMatch, DRACO-Gate is the retained deployable pre-rescue route point, while DRACO-ET and DRACO-Stack are frozen-candidate selector analyses. A dense 0–100% 3DLoMatch deployable threshold sweep was not present in the frozen workspace; the saved threshold-suite points and this limitation are explicitly recorded in the validation warnings and SQLite manifest.

Paired exact tests used in Supplementary Note S1.

Benchmark	Comparison	Total	Primary n (%)	Selected n (%)	Resc.	Regr.	Net	Exact p	Deploy.	Policy family
3DLoMatch	PointDSC+FCGF primary vs DRACO-Bundle	1781	762 (42.78)	849 (47.67)	127	40	87	9.45e-12	yes	deployable_pre_rescue_gate
3DLoMatch	PointDSC+FCGF primary vs DRACO-Gate	1781	762 (42.78)	855 (48.01)	150	57	93	7.75e-11	yes	deployable_pre_rescue_gate
3DLoMatch	PointDSC+FCGF primary vs DRACO-ET	1781	762 (42.78)	919 (51.60)	177	20	157	1.32e-32	no	frozen_candidate_selector
3DLoMatch	PointDSC+FCGF primary vs DRACO-Stack	1781	762 (42.78)	927 (52.05)	177	12	165	8.30e-39	no	frozen_candidate_selector
3DMatch	PointDSC+FCGF primary vs DRACO-Route	1623	1411 (86.94)	1442 (88.85)	39	8	31	5.54e-06	yes	deployable_pre_rescue_gate
3DMatch	PointDSC+FCGF primary vs RegTR counterfactual	1623	1411 (86.94)	1474 (90.82)	109	46	63	4.47e-07	no	always_on_rescue_counterfactual

Note: Exact p-values are two-sided binomial/McNemar-style tests over discordant pairs. Resc. = primary-fail/selected-success; Regr. = primary-success/selected-fail.

Supplementary Note S2. Qualitative success, regression, missed-rescue, and no-gain cases

The qualitative audit intentionally includes boundary cases rather than only positive examples. It records a successful rescue, a routed regression, a missed rescue outside the routed scenes, an unchanged failure, a net-negative always-on expansion that the bounded route avoids, and a minimal-effect hotel-family case.

Qualitative boundary cases used in Supplementary Note S2.

Case type	Scene	Pair	Primary/selected success	Primary RE/TE	Selected RE/TE	Runtime P/S ms	Policy	Explanation
successful rescue	studyroom2	cloud_bin_8.ply -> cloud_bin_51.ply	no/yes	10.91/0.0526	0.65/0.0030	584.26/7499.80	DRACO-Route	Primary fails, routed selection succeeds.
routed regression	studyroom2	cloud_bin_36.ply -> cloud_bin_39.ply	yes/no	2.58/0.0342	92.72/0.0432	717.05/8725.16	DRACO-Route	Primary succeeds, routed selection regresses.
missed rescue	redkitchen	cloud_bin_11.ply -> cloud_bin_28.ply	no/no	85.32/0.6501	85.32/0.6501	576.39/576.39	DRACO-Route	Deployable route stayed on primary, but always-on RegTR would have rescued the pair.
no-gain / unchanged failure	redkitchen	cloud_bin_15.ply -> cloud_bin_21.ply	no/no	117.87/0.5541	117.87/0.5541	468.24/468.24	DRACO-Route	Both routed output and always-on rescue remain strict failures.
net-negative expansion	redkitchen	cloud_bin_0.ply -> cloud_bin_32.ply	yes/yes	4.14/0.0141	4.14/0.0141	1017.80/1017.80	DRACO-Route	Always-on rescue would flip a valid primary alignment into failure, so bounded routing avoids a net-negative expansion.
minimal rescue effect scene	hotel_uc	cloud_bin_0.ply -> cloud_bin_1.ply	yes/yes	1.57/0.0112	1.57/0.0112	1197.51/1197.51	DRACO-Route	Hotel-family scene with little or no rescue benefit under the deployable route.

Note: RE/TE reports rotation error in degrees and translation-error ratio. Runtime P/S reports primary and selected runtime in milliseconds.

Supplementary Note S3. Redwood public-transfer audit

Redwood is retained as public-transfer validation and as a diagnostic boundary check. The manuscript's promotable transfer claim is that PointDSC+FCGF improves on the PointDSC+FPFH Redwood reference in the main table. The routed Redwood scene-gate probe is not promoted as routed superiority evidence because it adds only a marginal success gain over the PointDSC+FCGF primary row and remains weaker than the diagnostic oracle-like counterfactuals.

Redwood public-transfer and diagnostic route audit.

Policy	Family	Total	Activated	Act. %	Success n (%)	Resc.	Regr.	Net	Median	Mean	P95	Deploy.
Redwood PointDSC+FCGF primary	primary_baseline	1256	0	0.00	179 (14.25)	0	0	0	1196.10	1268.99	2114.86	no
Redwood Scene Gate	deployable_pre_rescue_gate	1256	269	21.42	182 (14.49)	3	0	3	1313.55	1527.81	3017.26	yes
Redwood Rescue On Failure	oracle_like_counterfactual	1256	24	1.91	203 (16.16)	24	0	24	1205.98	1293.59	2213.04	no
Redwood Oracle	oracle_like_counterfactual	1256	611	48.65	203 (16.16)	24	0	24	1659.27	1862.78	3601.52	no

Note: The scene gate is deployable but diagnostic; rescue-on-failure and oracle rows are counterfactual upper-bound checks.

Supplementary Note S4. Cue-level routing audits, matched CoFiNet evidence, missing-resource notes, and SQLite manifest

Cue timing is separated from candidate-side analysis. Rows with *Available before rescue = yes* are admissible for a deployable pre-rescue trigger. Rows with *Candidate-side = yes* depend on already materialized candidate outputs and are therefore reported only as frozen-candidate selector evidence.

Cue-level routing audit for Supplementary Note S4.

Bench.	Policy	Feature	Family	Before rescue	Candidate side	Threshold/rule	Normalization	Decision effect	Notes
3DLoMatch	DRACO-Bundle	predicted_positive_ratio	primary trace	yes	no	scene-specific gates: redkitchen<0.07; studyroom2<0.05; erika<0.06	native ratio, no extra scaling	Positive correspondence count divided by correspondence count in the saved primary trace.	Single-rescue scene bundle; optional predicted_positive cap retained in saved config.
3DLoMatch	DRACO-ET	candidate_route_runtime_ms	primary + self + candidate-side + categorical	no	yes	600 trees; 5 pair-hash folds; global margin 0.01 plus 8 grouped overrides	no additional scaling applied to the saved ET export	Frozen Extra-Trees selector over saved candidate pool; includes admissible primary cues plus candidate-side raw traces and delta/ratio features.	Promoted only as frozen-candidate selector analysis, not as a generic pre-routing trigger.
3DLoMatch	DRACO-ET	candidate_selected_probability	primary + self + candidate-side + categorical	no	yes	600 trees; 5 pair-hash folds; global margin 0.01 plus 8 grouped overrides	no additional scaling applied to the saved ET export	Frozen Extra-Trees selector over saved candidate pool; includes admissible primary cues plus candidate-side raw traces and delta/ratio features.	Promoted only as frozen-candidate selector analysis, not as a generic pre-routing trigger.
3DLoMatch	DRACO-ET	candidate_selector_margin	primary + self + candidate-side + categorical	no	yes	600 trees; 5 pair-hash folds; global margin 0.01 plus 8 grouped overrides	no additional scaling applied to the saved ET export	Frozen Extra-Trees selector over saved candidate pool; includes admissible primary cues plus candidate-side raw traces and delta/ratio features.	Promoted only as frozen-candidate selector analysis, not as a generic pre-routing trigger.
3DLoMatch	DRACO-ET	corr_count	primary + self + candidate-side + categorical	no	yes	600 trees; 5 pair-hash folds; global margin 0.01 plus 8 grouped overrides	no additional scaling applied to the saved ET export	Frozen Extra-Trees selector over saved candidate pool; includes admissible primary cues plus candidate-side raw traces and delta/ratio features.	Promoted only as frozen-candidate selector analysis, not as a generic pre-routing trigger.
3DLoMatch	DRACO-ET	dataset	primary + self + candidate-side + categorical	no	yes	600 trees; 5 pair-hash folds; global margin 0.01 plus 8 grouped overrides	no additional scaling applied to the saved ET export	Frozen Extra-Trees selector over saved candidate pool; includes admissible primary cues plus candidate-side raw traces and delta/ratio features.	Promoted only as frozen-candidate selector analysis, not as a generic pre-routing trigger.
3DLoMatch	DRACO-ET	delta_*_vs_primary	primary + self + candidate-side + categorical	no	yes	600 trees; 5 pair-hash folds; global margin 0.01 plus 8 grouped overrides	no additional scaling applied to the saved ET export	Frozen Extra-Trees selector over saved candidate pool; includes admissible primary cues plus candidate-side raw traces and delta/ratio features.	Promoted only as frozen-candidate selector analysis, not as a generic pre-routing trigger.
3DLoMatch	DRACO-ET	inference_ms	primary + self + candidate-side + categorical	no	yes	600 trees; 5 pair-hash folds; global margin 0.01 plus 8 grouped overrides	no additional scaling applied to the saved ET export	Frozen Extra-Trees selector over saved candidate pool; includes admissible primary cues plus candidate-side raw traces and delta/ratio features.	Promoted only as frozen-candidate selector analysis, not as a generic pre-routing trigger.
3DLoMatch	DRACO-ET	matching_ms	primary + self + candidate-side + categorical	no	yes	600 trees; 5 pair-hash folds; global margin 0.01 plus 8 grouped overrides	no additional scaling applied to the saved ET export	Frozen Extra-Trees selector over saved candidate pool; includes admissible primary cues plus candidate-side raw traces and delta/ratio features.	Promoted only as frozen-candidate selector analysis, not as a generic pre-routing trigger.
3DLoMatch	DRACO-ET	predicted_positive	primary + self + candidate-side + categorical	no	yes	600 trees; 5 pair-hash folds; global margin 0.01 plus 8 grouped overrides	no additional scaling applied to the saved ET export	Frozen Extra-Trees selector over saved candidate pool; includes admissible primary cues plus candidate-side raw traces and delta/ratio features.	Promoted only as frozen-candidate selector analysis, not as a generic pre-routing trigger.
3DLoMatch	DRACO-ET	predicted_positive_ratio	primary + self + candidate-side + categorical	no	yes	600 trees; 5 pair-hash folds; global margin 0.01 plus 8 grouped overrides	no additional scaling applied to the saved ET export	Frozen Extra-Trees selector over saved candidate pool; includes admissible primary cues plus candidate-side raw traces and delta/ratio features.	Promoted only as frozen-candidate selector analysis, not as a generic pre-routing trigger.
3DLoMatch	DRACO-ET	prediction_runtime_ms	primary + self + candidate-side + categorical	no	yes	600 trees; 5 pair-hash folds; global margin 0.01 plus 8 grouped overrides	no additional scaling applied to the saved ET export	Frozen Extra-Trees selector over saved candidate pool; includes admissible primary cues plus candidate-side raw traces and delta/ratio features.	Promoted only as frozen-candidate selector analysis, not as a generic pre-routing trigger.
3DLoMatch	DRACO-ET	ratio_*_vs_primary	primary + self + candidate-side + categorical	no	yes	600 trees; 5 pair-hash folds; global margin 0.01 plus 8 grouped overrides	no additional scaling applied to the saved ET export	Frozen Extra-Trees selector over saved candidate pool; includes admissible primary cues plus candidate-side raw traces and delta/ratio features.	Promoted only as frozen-candidate selector analysis, not as a generic pre-routing trigger.
3DLoMatch	DRACO-ET	runtime_ms	primary + self + candidate-side + categorical	no	yes	600 trees; 5 pair-hash folds; global margin 0.01 plus 8 grouped overrides	no additional scaling applied to the saved ET export	Frozen Extra-Trees selector over saved candidate pool; includes admissible primary cues plus candidate-side raw traces and delta/ratio features.	Promoted only as frozen-candidate selector analysis, not as a generic pre-routing trigger.
3DLoMatch	DRACO-ET	self_*	primary + self + candidate-side + categorical	no	yes	600 trees; 5 pair-hash folds; global margin 0.01 plus 8 grouped overrides	no additional scaling applied to the saved ET export	Frozen Extra-Trees selector over saved candidate pool; includes admissible primary cues plus candidate-side raw traces and delta/ratio features.	Promoted only as frozen-candidate selector analysis, not as a generic pre-routing trigger.

Continued on next page

Bench.	Policy	Feature	Family	Before rescue	Candidate side	Threshold/rule	Normalization	Decision effect	Notes
3DLoMatch	DRACO-Gate	corr_count	primary trace	yes	no	representative retained rules: studyroom2->RegTR if ratio<0.06; redkitchen->PredatorBundle if predicted_positive<=300 and corr_count<=8000	native units, no extra scaling	Primary-pass ratio/count cues used by the multi-rescue scene gate.	Scene identity chooses both rescue target and cue thresholds.
3DLoMatch	DRACO-Gate	predicted_positive	primary trace	yes	no	representative retained rules: studyroom2->RegTR if ratio<0.06; redkitchen->PredatorBundle if predicted_positive<=300 and corr_count<=8000	native units, no extra scaling	Primary-pass ratio/count cues used by the multi-rescue scene gate.	Scene identity chooses both rescue target and cue thresholds.
3DLoMatch	DRACO-Gate	predicted_positive_ratio	primary trace	yes	no	representative retained rules: studyroom2->RegTR if ratio<0.06; redkitchen->PredatorBundle if predicted_positive<=300 and corr_count<=8000	native units, no extra scaling	Primary-pass ratio/count cues used by the multi-rescue scene gate.	Scene identity chooses both rescue target and cue thresholds.
3DLoMatch	DRACO-Stack	candidate_selected_probability	candidate-side overlay	no	yes	global overlay margin 0.01; margins 0.01-0.08 tie on the same best strict-success plateau	native probabilities, no extra scaling	Overlay ET selector replaces the incoming ET output only when the overlay candidate beats it by the retained margin.	Promoted 3DLoMatch headline row; post-hoc/frozen-candidate rather than admissible pre-routing.
3DLoMatch	DRACO-Stack	candidate_selector_margin	candidate-side overlay	no	yes	global overlay margin 0.01; margins 0.01-0.08 tie on the same best strict-success plateau	native probabilities, no extra scaling	Overlay ET selector replaces the incoming ET output only when the overlay candidate beats it by the retained margin.	Promoted 3DLoMatch headline row; post-hoc/frozen-candidate rather than admissible pre-routing.
3DLoMatch	DRACO-Stack	dataset	candidate-side overlay	no	yes	global overlay margin 0.01; margins 0.01-0.08 tie on the same best strict-success plateau	native probabilities, no extra scaling	Overlay ET selector replaces the incoming ET output only when the overlay candidate beats it by the retained margin.	Promoted 3DLoMatch headline row; post-hoc/frozen-candidate rather than admissible pre-routing.
Redwood	Redwood scene gate	predicted_positive	primary trace	yes	no	livingroom2-simulated: predicted_positive_ratio<0.03 and predicted_positive<=200; office1-simulated: predicted_positive_ratio<0.04	native units, no extra scaling	Low-cost cues used by the retained Redwood diagnostic gate.	Reported as diagnostic only because deployable gain is marginal.
Redwood	Redwood scene gate	predicted_positive_ratio	primary trace	yes	no	livingroom2-simulated: predicted_positive_ratio<0.03 and predicted_positive<=200; office1-simulated: predicted_positive_ratio<0.04	native units, no extra scaling	Low-cost cues used by the retained Redwood diagnostic gate.	Reported as diagnostic only because deployable gain is marginal.
3DMatch	DRACO-Route	dataset (scene key)	scene metadata	yes	no	studyroom2 or erika => RegTR; all other scenes stay on PointDSC+FCGF	categorical, no scaling	Route to RegTR when the saved 3DMatch scene key belongs to the fixed bundle.	No score threshold; 369/1623 pairs routed (22.74%).

Note: Feature groups such as self_* and delta_*_vs_primary represent the grouped feature families stored in the frozen route audit.

Matched CoFiNet baseline reconstruction used in Supplementary Note S4.

Benchmark	Baseline	Matched protocol	Total	Strict success	Median RE	Median TE	Median rt.	Checkpoint/source	Notes
3DLoMatch	CoFiNet	saved est.log reconstruction on frozen 1781-pair pool	1781	960 (53.90)	4.2647	0.0285	–	draco_align_project/third_party/CoFiNet/weights/best_3dmatch.pth; est_traj_setting=5000	{"runtime_availability": "Saved CoFiNet est_traj logs do not expose per-pair runtime; runtime columns are NULL.", "selected_setting": "5000"}

Note: Runtime columns are unavailable because the saved CoFiNet trajectory logs do not store per-pair runtime.

Missing-resource notes for Supplementary Note S4.

Item	Expected purpose	Missing reason	Consequence	Recommended action
CoFiNet matched baseline per-pair runtime	Matched baseline runtime distribution	Saved CoFiNet est.log trajectories do not store per-pair runtime measurements.	CoFiNet matched runtime columns are NULL even though accuracy metrics are fully reconstructed.	Rerun CoFiNet under a timed pairwise wrapper if matched runtime is required.
GeoTransformer matched 3DLoMatch baseline	Priority full-pool matched 3DLoMatch SOTA baseline	Workspace only contains smoke-level GeoTransformer scored outputs (2 pairs) rather than a full 1781-pair result bundle.	GeoTransformer cannot be used as the matched same-pool baseline in this revision database.	Restore or rerun a full GeoTransformer 3DLoMatch 1781-pair evaluation before promoting it as matched evidence.
OverlapPredator matched 3DLoMatch baseline	Priority full-pool matched 3DLoMatch SOTA baseline reconstruction	Saved raw artifact has 1781 metadata rows but only 464 transformation outputs; remaining pairs fail with missing feature files in the saved snapshot.	OverlapPredator cannot be reconstructed as a complete 1781-pair matched baseline from the frozen workspace.	Restore the missing OverlapPredator snapshot feature files or rerun the full all8 extraction/inference stage.
SC2-PCR matched 3DLoMatch baseline	Fallback matched 3DLoMatch SOTA baseline option	No SC2-PCR script, checkpoint, or saved result artifact was found in the workspace scan.	SC2-PCR could not be reconstructed or run.	Add the SC2-PCR repository checkout plus checkpoint/results if this baseline is required.
Torch-enabled deep-model runtime environment	Direct reruns of GeoTransformer / Predator / SC2-PCR pairwise inference from the current shell	Default python environment available to this task does not provide torch.	Heavy deep-model reruns were not executed from scratch; reconstruction relied on saved artifacts wherever possible.	Activate the original training/inference environment or install torch-compatible dependencies before rerunning heavy baselines.

Note: These notes disclose unavailable full matched reruns and explain why the manuscript does not promote them as synchronized evidence.

SQLite reproducibility manifest for Supplementary Note S4.

Manifest field	Value
Database file	revision_experiment_data.sqlite
SHA-256	c5d5abf4138184abe49cd1e0fb7fdac1ee9c961d6daa6117ecfdcea07b4e35a7
File size	16,310,272 bytes
per_pair_results rows	18798
matched_sota_baseline_per_pair rows	1781
validation_checks	47 total; 47 PASS; 0 FAIL
validation_warnings	3DLoMatch_route_sensitivity_scope: Only the saved deployable threshold suite and the saved final scene_gate point were available; no dense 0-100 threshold sweep artifact existed in the frozen workspace.
Table row counts	confidence_intervals=12; file_inventory=4223; matched_sota_baseline_per_pair=1781; matched_sota_baseline_summary=1; missing_resources=5; paired_significance=6; per_pair_results=18798; policy_activation_regression=12; qualitative_case_spectrum=6; route_rate_sensitivity=9; routing_feature_thresholds=24; run_metadata=9; runtime_distribution=12; validation_checks=47; validation_warnings=1

Note: The SQLite database is the authoritative row-level evidence artifact accompanying the revision package.
